# Association of Exosomal miR-34a with Markers of Dyslipidemia and Endothelial Dysfunction in Children and Adolescents with T1DM

**DOI:** 10.4274/jcrpe.galenos.2020.2020.0134

**Published:** 2020-11-25

**Authors:** Alshaymaa A. Ibrahim, Aliaa A. Wahby, Ingy Ashmawy, Rehan M. Saleh, Hend Soliman

**Affiliations:** 1 National Research Centre, Department of Clinical and Chemical Pathology, Cairo, Egypt; 2 National Research Centre, Department of Community, Cairo, Egypt; 3 Cairo University Faculty of Medicine, New Children Hospital, Cairo, Egypt

**Keywords:** miR-34a, dyslipidemia, endothelial dysfunction, type 1 diabetes mellitus, endoglin, intracellular adhesion molecule

## Abstract

**Objective:**

Dyslipidemia and endothelial dysfunction are common disorders and major causative factors for atherosclerosis in patients with type 1 diabetes mellitus (T1DM). However, their pathophysiology in young patients with T1DM is still under evaluated. We aimed, for the first time, to assess the expression of exosomal micro-RNA 34a (miR-34a) in serum of children and adolescents with T1DM and correlate this expression with markers of dyslipidemia and endothelial dysfunction.

**Methods:**

The study included 120 T1DM patients and 100 control subjects. Assessment of miR-34a was performed using quantitative real-time polymerase chain reaction. Lipid profile was assessed on an automated analyzer and serum endoglin and intracellular adhesion molecule (ICAM) concentrations were measured using immunometric methods.

**Results:**

Relative expression of miR-34a and serum endoglin and ICAM concentrations were higher in patients than controls (p=0.001) and in patients with dyslipidemia (42 patients) compared to patients without dyslipidemia (78 patients) (p=0.01). Linear regression analysis revealed a strong independent association between exosomal miR-34a expression and total cholesterol, low-density lipoprotein, serum endoglin and serum ICAM after adjustment for other cofactors. The utility of miR-34a as an indicator for associated dyslipidemia was tested using receiver operator characteristic curve analysis which revealed area under the curve: 0.73 with confidence interval: 0.63-0.83 and p=0.001.

**Conclusion:**

This was the first study to show the altered expression of exosomal miR-34a among children and adolescents with T1DM. Moreover, association of miR-34a with markers of dyslipidemia and endothelial dysfunction was identified, suggesting that it could play a role in regulation of lipid metabolism and endothelial function in T1DM.

**What is already known on this topic?**

Dyslipidemia and endothelial dysfunction are common disorders and major predisposing factors for atherosclerosis and cardiovascular diseases in patients with type 1 diabetes mellitus (T1DM). However, their pathophysiology in children and adolescents with T1DM is still under evaluated.

**What this study adds?**

Association of exosomal micro-RNA 34a serum expression with markers of dyslipidemia and endothelial dysfunction was identified in children and adolescents with T1DM, suggesting its role in regulation of lipid metabolism and endothelial function in T1DM.

## Introduction

Type 1 diabetes mellitus (T1DM) is a complex, multifactorial, autoimmune illness and continues to increase in prevalence among children and adolescents (1). T1DM affects about 35 million persons all over the world with annual increase ranging between 3-5% (2).

Dyslipidemia and endothelial dysfunction are very common metabolic abnormalities in these patients (3,4). Both are major causative factors for atherosclerosis which is a major precursor of cardiovascular disease (CVD), the leading cause for morbidity and mortality in T1DM (5). However, the pathophysiology underlying the occurrence of dyslipidemia and endothelial dysfunction in young patients with T1DM remains unclear. It is suggested that the pathogenesis involves an interaction between genetic, environmental and eventually epigenetic factors. Epigenetic factors, including microRNAs, not only represent a key for understanding the complexity of vascular diseases in these patients but also represent a new field of investigation to discover new diagnostic and prognostic markers (6).

MicroRNA (miRNA, miR) is a class of small, noncoding RNA, which play a significant role in regulating gene expression. Therefore, miRNAs could contribute to the pathogenesis of a number of different physiological and pathological processes (7). Exosomes are nanovesicles originating from all cells, whether healthy or diseased, and can be found in all body fluids. The lipid bilayer surrounding exosomes enable exosomal-enclosed miRNAs to be stably expressed in body fluids much more so than free circulating miRNAs. Consequently, recent studies have focused on exosomal-enclosed miRNAs as contributing factors in various human diseases (8).

Accumulating data have demonstrated that miR-34a contributes to b-cell apoptotic pathways, suggesting a role in T1DM (9). Among different miRNA candidates, miR-34a is of special interest regarding lipid metabolism and endothelial function because of its interaction with many genes involved in both pathways (10). Genomic data for exosomal miR-34a were retrieved from the extracellular vesicles miRNA database (11,12), while the predicted miRNA target genes were analyzed by using DIANA-miRPath v1.1 webserver (13). Accordingly, its role in adipogenesis, atherosclerosis progression, inflammation and CVD development and progression, has been suggested.

The current study aimed, for the first time, to assess the expression of exosomal miR-34a in the serum of children and adolescents with T1DM and to evaluate the association between exosomal miR-34a expression and markers of dyslipidemia, such as total cholesterol (TC), low-density lipoprotein cholesterol (LDL-C), high-density lipoprotein cholesterol (HDL-C) and triglycerides (TG), as well as the serum levels of markers of endothelial dysfunction, including serum endoglin and intracellular adhesion molecule (ICAM).

## Methods

This study is a pilot cross-sectional study. A total of 120 T1DM patients, with disease duration more than five years, were randomly selected from Diabetes, Endocrine and Metabolism Pediatric Unit, Pediatric Department, Faculty of Medicine, Cairo University, with respect to inclusion and exclusion criteria (see below). Confirmation of T1DM diagnosis was based on criteria of American Diabetes Association (ADA) (14).

- Exclusion criteria in the current study included: any type of diabetes other than T1DM; T1DM with any microvascular complication; hypertension; heart, liver, or renal insufficiency; acute diabetic complications; systemic inflammatory diseases; other endocrine disorders; neoplastic disorders; and family history of dyslipidemic diseases.

- One hundred age and sex matched healthy subjects served as control group and were recruited from the National Research Centre (NRC) during regular check-up.

- The included patients were subsequently categorized into patients with dyslipidemia (42 patients) and patients without dyslipidemia (78 patients). According to the ADA, dyslipidemia was defined by the presence of one or more of the following criteria: TC ≥200 mg/dL, LDL-C ≥130 mg/dL, HDL-C ≤35 mg/dL, and TG ≥150 mg/dL (15).

- Informed consent was obtained from each participant. This study was approved by the Ethics Committee of the NRC, under approval number 16/285, in accordance with the Declaration of Helsinki 2015.

All participants were subjected to full history taking and full clinical examination.

### Biochemical Analysis

- Venous blood samples were collected from all subjects after 12 hours of overnight fast.  Serum was separated and parameters of dyslipidemia including, TC, TG, HDL and LDL levels were quantified using Erba Mannheim XL300 Chemistry Analyzer (ERBA Diagnostics Mannheim, Medical EXPO, India).

- Markers of endothelial dysfunction including serum endoglin and ICAM concentrations were assessed using enzyme linked immunosorbent assay sandwich technique (Quantikine, R&D Systems, Minneapolis, USA).

Assessment of exosomal miR-34a was done by quantitative reverse transcriptase real-time polymerase chain reaction (qRT PCR) technique:

Exosomes were isolated from eight hundred microliters of serum according to exoRNeasy Serum/Plasma midi kit (Qiagen, Hilden, Germany). The characteristics of isolated exosomes were confirmed by transmission electron microscopy (16). Isolation, purification and elution of exosomal RNA was done according to exoRNeasy Serum/Plasma midi kit’s protocol and 3.5 microns of synthetic spike in control Cel-miR-39 was added to each sample as the internal control. Concentration of isolated RNA was assessed using a NanoDrop 2000c spectrophotometer (ThermoFisher Scientific, Waltham, Massachusetts, USA). Complementary DNA (cDNA) was synthesized using a miScript reverse transcription kit (Qiagen, Hilden, Germany) and then all specimens were stored at -80 °C. Quantitative PCR was run on Applied technologies, Stratagene Mx3000P using miScript SYBR green PCR kit (Qiagen, Hilden, Germany) for detection of miR-34a (ID: MS00003318). The relative expression of miRNA was described as fold change using the calculated formula; 2-ΔΔCT. Methods have been described in detail elsewhere (8).

### Statistical Analysis

Patients’ clinical and laboratory quantitative and qualitative data were presented as mean±standard deviation and frequencies respectively, while the levels of relative miR-34 expression were presented as median [interquartile range (IQR)]. Non-parametric tests were used to evaluate expression difference of miR-34a between patients and healthy controls and between patients’ groups. To assess the relationships between exosomal miR-34a and different patients’ parameters, Pearson’s correlation and linear regression were performed. The utility of miR-34a as an indicator for associated dyslipidemia among patients was tested using receiver operating curve (ROC) technique and area under the curve (AUC) was calculated. All tests were two-sided and a p<0.05 was considered statistically significant.

## Results

The clinical, demographic and routine laboratory test results for patients and healthy controls are presented in Table 1. Both patients and controls were matched for age, gender and body mass index (BMI). Glycated hemoglobin (HbA1c), TC, TGs and LDL were significantly increased in patients compared to controls, while HDL showed no significant difference between the two groups.

**Figure 1:**
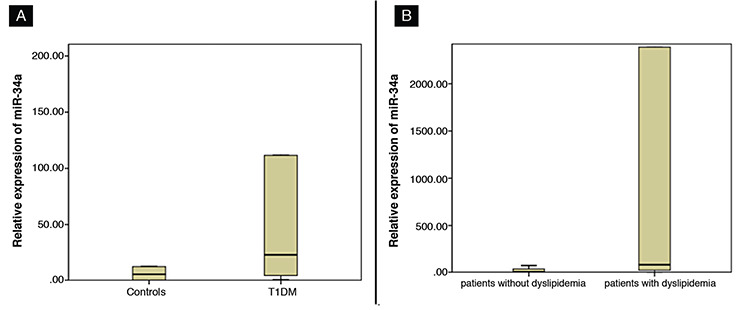
Expression of exosomal micro-RNA 34a (miR-34a) among studied groups. A) Shows significant higher expression of miR-34a in patients with type 1 diabetes mellitus [median: 22.6, interquartile range (IQR): 4.2-111.7] compared to healthy controls (median: 6, IQR: 0.1-12). B) Shows increased expression of exosomal miR-34a in patients with dyslipidemia (median: 78, IQR: 18.1-2388) compared to patients without dyslipidemia (median: 4.8, IQR: 3.7-34.2) miR-34a: micro-RNA 34a, T1DM: type 1 diabetes mellitus

Regarding miRNA expression, miR-34a showed significant higher expression among T1DM patients (median: 22.6, IQR: 4.2-111.7 fold change) than healthy controls (median: 6, IQR: 0.1-12 fold change) (p=0.001) (Figure 1). Frequency of patients with miR-34a overexpression, defined as expression more than  two-fold change, among T1DM patients was 90%. Association of miR-34a expression and T1DM was confirmed using logistic regression analysis after adjustment for age, gender and BMI (p=0.01).

Comparison between patients regarding associated dyslipidemia showed that the relative expression of exosomal miR-34a was higher in patients with dyslipidemia (median: 78; IQR: 18.1-2388 fold change) compared to patients without dyslipidemia (median: 4.8; IQR: 3.7-34.2 fold change) (p=0.001) (Figure 1). There was no significant difference between patients with dyslipidemia and those without dyslipidemia regarding age, gender, BMI and HbA1c. Disease duration, serum TC, TGs and LDL-C were significantly higher in patients with dyslipidemia compared to patients without dyslipidemia, while HDL showed no significant difference between the two groups (Table 2). The most common disorder of dyslipidemia was hypercholesterolemia (95%). Prevalence of high TG, low HDL-C and high LDL-C was 9.5%, 12% and 45% respectively.

Levels of serum endoglin and serum ICAM were significantly higher in patients with T1DM than healthy subjects (p=0.01 and p=0.001, respectively) (Table 1) and higher in T1DM patients with dyslipidemia compared to patients without dyslipidemia (p=0.01) (Table 2). Serum endoglin and serum ICAM showed no significant correlation with age, BMI, disease duration, glycated hemoglobin and parameters of the lipid profile (Table 3).

**Figure 2:**
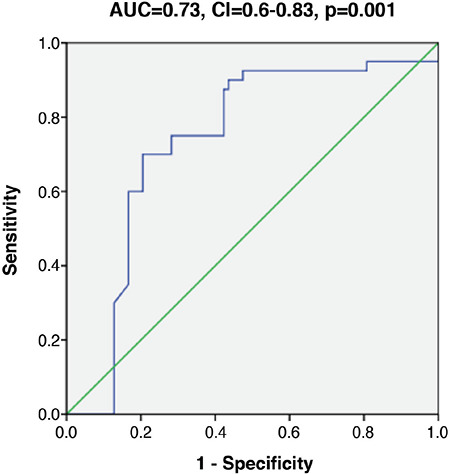
Receiver operating curve (ROC) curve of exosomal micro-RNA 34a (miR-34a) in associated dyslipidemia in type 1 diabetes mellitus. ROC curve showed the utility of miR-34a as an indicator of associated dyslipidemia. Area under the curve: 0.73 with confidence intervals: 0.63-0.83 (p=0.001) AUC: area under the curve, CI: confidence interval

Pearson’s correlation revealed positive correlation between miR-34a and both serum endoglin and serum ICAM concentration, but failed to demonstrate significant association between miR-34a with parameters of lipid profile (Table 4). Linear regression analysis was used to confirm the association of exosomal expression of miR-34a with parameters of lipid metabolism and endothelial dysfunction in patients with T1DM after adjustment for age, gender, BMI and disease duration. This analysis revealed a strong independent association between exosomal miR-34a with TC, serum endoglin and serum ICAM (Table 4). The utility of miR-34a as indicator for associated dyslipidemia was tested using ROC curve which revealed AUC: 0.73 with confidence intervals: 0.63-0.83 (p=0.001) (Figure 2).

**Figure 3:**
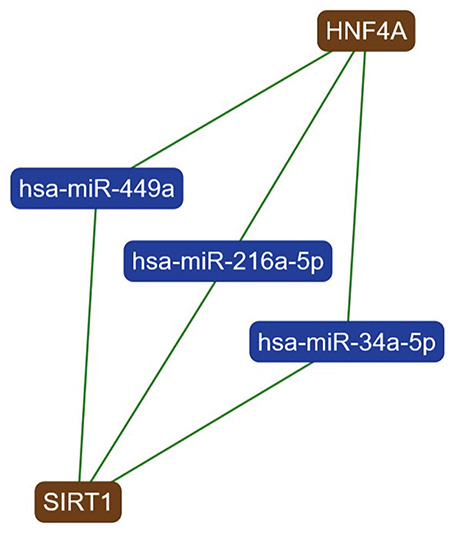
Interaction network of genes targeted by micro-RNA 34a (miR-34a) and playing a significant role in lipid metabolism. This analysis was done using (miRTargetLinkdatabase) (https://ccb-web.cs.uni-saarland.de/mirtargetlink/index.php) and retrieved that miR-34a is one of the three miRNAs that can target both *hepatocyte nuclear factor 4 (HNF4)* and the *sirtuin 1 (SIRT1)* genes (green line indicates a strong interaction)

**Figure 4:**
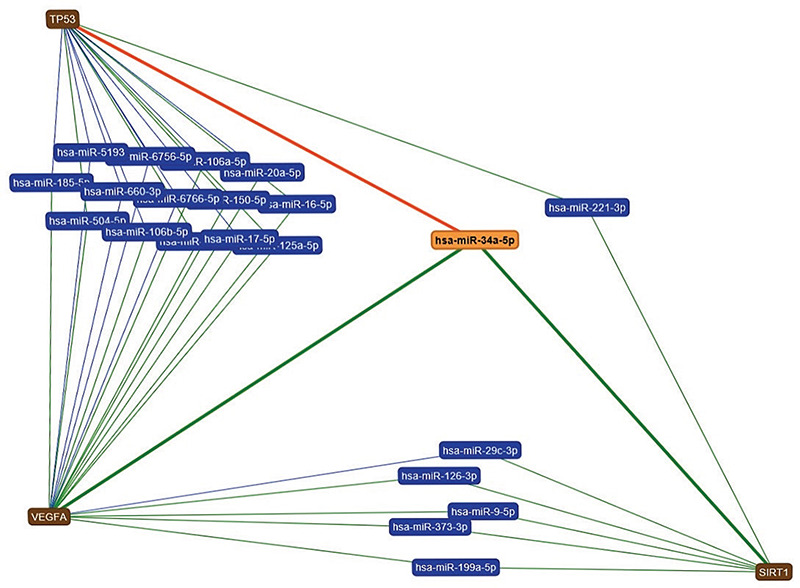
Interaction network of genes targeted by micro-RNA 34a (miR-34a) and playing a significant role in endothelial function. This analysis was done using (miRTargetLinkdatabase) (https://ccb-web.cs.uni-saarland.de/mirtargetlink/index.php) and retrieved that miR-34a is the only miRNA that targets the three major genes, vascular endothelial growth factor, *sirtuin 1 (SIRT1)* and *p53*, involved in endothelial function (green line indicates a strong interaction and red line indicates a weak interaction)

To validate and strengthen our results, the analysis of miRNAs that target different genetic pathways involved in lipid metabolism and endothelial function was done using https://ccb-web.cs.uni-saarland.de/mirtargetlink/index.php. This analysis retrieved miR-34a as one of the three miRNAs that can target both the *hepatocyte nuclear factor 4 (HNF4)* and *sirtuin 1 (SIRT1)* genes that have been identified as playing major roles in lipid metabolism (Figure 3); In addition miR-34a was the only miRNA that targeted the three major genes, vascular endothelial growth factor (VEGF), *SIRT1* and *p53*, involved in endothelial function (Figure 4) (17).

## Discussion

Pathogenesis of associated endothelial dysfunction and dyslipidemia in children and adolescents with T1DM is still under-evaluated. This is the first study to evaluate exosomal miR-34a expression in children and adolescents with T1DM and to correlate this expression with markers of dyslipidemia and endothelial dysfunction in the studied patients.

The role of miR-34a in the development of diabetes is under the spotlight. Accumulating data indicate that miR-34a plays significant roles in glucose sensing, insulin secretion, and increasing sensitivity of b cells to cytokine-induced apoptosis (18,19,20). In the current study, expression of miR-34a was increased in patients with T1DM compared to healthy subjects, suggesting its role in the pathogenesis of T1DM. This is in agreement with other studies that demonstrated over-expression of miR-34a in T1DM (21,22), especially in recent-onset T1DM, compared to high-risk and healthy children (23).

Dyslipidemia is a metabolic disorder commonly associated with T1DM, increasing the risk of CVD (5). In our study, the prevalence of dyslipidemia among patients with T1DM was 35%. El-Bakry et al (24) reported that 64% of Egyptian children and adolescents with T1DM showed association with dyslipidemia. In addition, the prevalence of dyslipidemia in children and adolescents with T1DM is reported to vary from 29% to 66% in cross sectional studies from different countries (25,26,27). The most common types of dyslipidemia among adolescents with T1DM also varies between hypercholesterolemia (24,28), high triglyceridemia (29,30) and high LDL-C (31,32). Differences in sample size, inclusion and exclusion criteria, degree of glycemic control and ethnicity might be the cause of this wide range of prevalences and difference in frequencies of dyslipidemia types in the previous studies.

MiR-34a is known to regulate TC and hepatic lipid metabolism through targeting and inhibition of expression of the NAD+-dependent lysine deacetylase *SIRT1*, the antiatherogenic mediator that regulates lipid metabolism and endothelial function (33). MiR-34a also down regulates *HNF4*, a gene which modulates the expression of other genes involved in lipid and glucose metabolism (34). In agreement with the previous data, we have demonstrated for the first time increased miR-34a expression in patients with dyslipidemia compared to those without dyslipidemia. Moreover, miR-34a overexpression was described in different diseases associated with dyslipidemia, particularly non-alcoholic fatty liver disease (33,35) and coronary artery disease (36) and this expression was correlated with disease severity. Linear regression analysis for our results revealed positive association between miR-34a and TC and LDL-C, while no correlation or association was detected between the miRNA and TG. Interestingly, Shen et al (20) reported positive correlation of miR-34a expression with LDL-C and negative correlation with TG in patients with T2DM. However, Xu et al (34) suggested an explanation when they showed that miR-34a regulates hepatic TGs via regulation of *HNF4* expression and its overexpression leads to accumulation of TGs in the liver and subsequently decreasing serum TGs.

Endothelial dysfunction precedes and promotes vascular inflammation and therefore atherosclerosis in T1DM and may be considered as an independent predictor of associated CVD (3). Endoglin (CD105) is a membrane glycoprotein located on the surface of vascular endothelial cells. Endoglin, acts as a receptor for transforming growth factor-beta (TGF-b) which is an important mediator for angiogenesis and responsible for keeping the vascular endothelium healthy (37). ICAM is a surface adhesion molecule, also expressed on vascular endothelial cells and also immune cells, which facilitates cell-to-cell interaction and recruitment of leucocytes to endothelium during inflammation (38). Shedding of endoglin and ICAM1 receptors into the systemic circulation during endothelial injury renders them potential circulatory markers of endothelial dysfunction (37). In this study, levels of serum endoglin and serum ICAM were highly elevated in patients with T1DM compared to healthy controls. In agreement with our results, there have been previous reports of elevated levels of serum endoglin and serum ICAM in patients with T1DM (38,39,40). Another interesting finding was the elevation of serum endoglin and serum ICAM in patients with dyslipidemia compared to patients without dyslipidemia, in agreement to El-Kassas et al (39), who reported significant positive correlations of endoglin with TC, TG and LDL-C in children with T1DM. In addition, serum endoglin and serum ICAM showed positive correlations with exosomal miR-34a expression in our study. This data was consistent with other reports, stating that miR-34a is one of the most important endothelial miRNAs which plays a significant role in maintaining endothelial cell function by targeting many genes including **p53**, VEGF and *SIRT1* (41). Moreover, TGF-b increases endoglin and can upregulate miR-34a, which subsequently promotes vascular inflammation by upregulating vascular cell adhesion molecule-1 and ICAM (10,42). Interestingly, it was reported that miR-34a deletion in the endothelium preserve endothelial functions in diabetic mice indicating the responsibility of miR-34a for diabetic vascular dysfunction (10).

### Study Limitations

The partial small sample size and the cross-sectional research design may be considered as limitations for this study.

## Conclusion

To the best of our knowledge, this is the first study to report the altered expression of exosomal miR-34a among children and adolescents withT1DM. Moreover, association of miR-34a with markers of dyslipidemia and endothelial dysfunction was demonstrated, suggesting a role for miR-34 in the epigenetic regulation of lipid metabolism and endothelial function in T1DM children and adolescents. More longitudinal studies with larger sample sizes and prospective design are warranted to investigate this association further. Future studies are recommended to identify the possible use of miR-34a as a therapeutic target in patients with T1DM and CVD.

## Figures and Tables

**Table 1:**
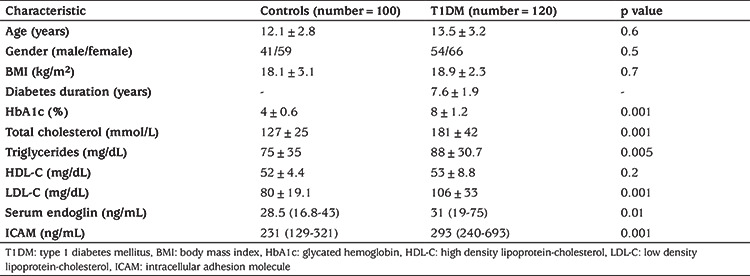
Clinical, demographic and biochemical laboratory data of patients and controls

**Table 2:**
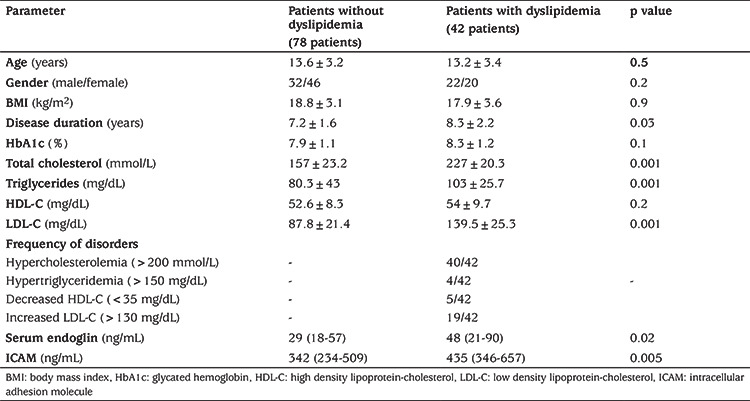
Comparison between patients with dyslipidemia and patients without dyslipidemia

**Table 3:**
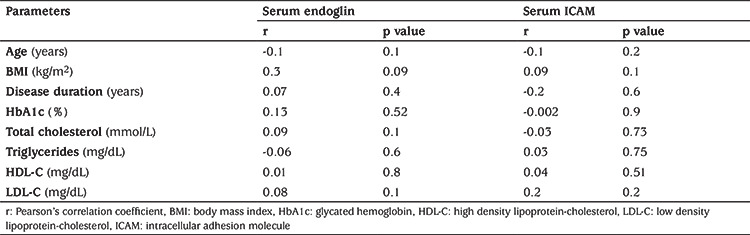
Pearson’s correlation of serum endoglin and serum intracellular adhesion molecule with different parameters in type 1 diabetes mellitus patients

**Table 4:**
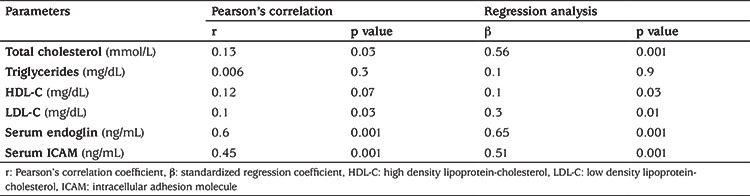
Pearson’s correlation and linear regression analysis of micro-RNA 34a with different parameters of dyslipidemia and endothelial dysfunction
